# The Effect of Calcium and Glucose Concentration on Corneal Epithelial Cell Lines Differentiation, Proliferation, and Focal Adhesion Expression

**DOI:** 10.1089/biores.2018.0036

**Published:** 2019-06-05

**Authors:** Sophia Masterton, Mark Ahearne

**Affiliations:** ^1^ Trinity Centre for Biomedical Engineering, Trinity Biomedical Science Institute, Trinity College Dublin, University of Dublin, Dublin, Ireland.; ^2^ Department of Mechanical and Manufacturing Engineering, School of Engineering, Trinity College Dublin, University of Dublin, Dublin, Ireland.

**Keywords:** calcium, cornea, epithelium, glucose

## Abstract

It is known that culture media composition can affect cell behavior, morphology, and gene expression. However, in the case of corneal epithelial cells, the combined role of calcium and glucose concentration in media has not previously been examined. In this study, a human immortalized corneal epithelial cell line was used to examine the effect of glucose and calcium concentrations on these cells. Cell metabolic activity, cell growth curve analysis, and relative gene and protein expression of proliferative marker extracellular related kinase (ERK) were used to study proliferation. Corneal epithelial stem cell marker NP63 and mature epithelial marker cytokeratin 3 (CK3) were analyzed by using reverse transcription polymerase chain reaction (RT-PCR) and immunocytochemistry. Focal adhesions were examined by using immunocytochemistry. Cells cultured in both low-glucose, high-calcium (LG-HC) media and high-glucose, low-calcium (HG-LC) media showed similar results in both RT-PCR and immunocytochemistry analysis. NP63 expression was significantly lower and CK3 expression was higher in these groups compared with cells cultured in commercial media. NP63 and CK3 expression was also analyzed by using immunocytochemistry, which confirmed these findings. The high-glucose, high-calcium-fed cells showed the lowest expression of all markers and no gene expression of CK3. This was deemed the most unsuitable media formulation for this cell line. Focal adhesion expression was the lowest in the high-calcium, high-glucose-fed cells, with the most even distribution of this among the commercial media group. Overall, this study showed that varying glucose and calcium concentrations can have significant effects on differentiation, proliferation, focal adhesions, and metabolic activity of this cell line. It seems that an LG-HC and HG-LC formulation were interchangeable with similar proliferative and differentiation effects.

## Introduction

The corneal epithelium is the stratified squamous outer layer of the cornea that is maintained by limbal epithelial stem cells located in crypts along the cornea-scleral border.^1^ Severe damage to the epithelium can cause blindness and require either keratoplasty from donor tissue or the transplantation of limbal stem cells in cases of limbal stem cell deficiency. These cells may also be expanded *in vitro* and undergo epithelial differentiation to generate cell sheets that are suitable for transplantation.^2^ Although there has been much research undertaken using different culture media, further work is still required to find an optimal media formulation expanding the cell population and promoting epithelial differentiation.

Two factors that can influence the behavior of corneal epithelial and limbal cells are the glucose and calcium concentrations within the culture media. Glucose concentration has been associated with changes in matrix metalloproteinase activity,^3^ immune response,^4^ and growth factor signaling^5^ in corneal epithelial cells. Calcium concentration has been shown to affect both the proliferation and differentiation of mice corneal epithelial cells *in vitro*.^6^ Although the role of each of these two important reagents has been examined, their reciprocal role in cell behavior has not previously been explored. Therefore, the aim of this study was to examine the combined role that calcium and glucose concentration have in the behavior of corneal epithelial cells. This information is particularly important when trying to determine the optimal media conditions needed for expanding or differentiating these cells.

This study uses a human corneal epithelial cell line isolated from the limbus that is used widely in corneal epithelial research^7–9^ to determine how different glucose and calcium concentrations in combination affect the cells ability to proliferate and differentiate. The use of a cell line in this study removes the potential for donor variation. Proliferation was determined by examining the proliferative marker extracellular related kinase (ERK) at both the gene and protein level in addition to measuring metabolic activity and cell growth over 7 days.

Differentiation was characterized by examining epithelial stem cell marker NP63 and mature corneal epithelial marker cytokeratin 3 (CK3). NP63 is a protein expressed by stem cells located in the limbus but is lost when these cells migrate out from this region into the epithelium and differentiate into epithelial cells.^10,11^ CK3 is not expressed by limbal stem cells but after epithelial differentiation, the expression of this marker can be detected. CK3 is present in the central corneas superficial epithelial cells and absent from the limbus.^10^ In addition, the affects that the media had on cellular focal adhesions, which are critical to cellular communication and migration, were also examined.

## Materials and Methods

### Cell culture

A vial of an immortalized human corneal epithelial cell line (hTCEpi; Evercyte) was used in all the experiments in this study. The cells ectopically express the catalytic subunit of human telomerase. The cell line was thawed by pre-warming in hand and transferring the contents to a tube filled with 9-mL pre-warmed Keratinocyte Growth Medium-2 (KGM-2; PromoCell). The cells were centrifuged at 170 *g* for 5 min, and the pellet was resuspended in 1-mL media. This was transferred to a T25 culture flask containing 6-mL media and incubated at 37°C. The culture medium was changed after 24 h and passaged if already confluent.

Cells were seeded at 5000 cells/cm^2^ into cell culture plates. Cells were used between passage 4 and 6 for the purpose of testing media formulations. The cells were fed 3 days a week with corneal epithelial media containing Dulbecco's modified Eagle's medium (DMEM) and Ham's F12 medium in a 3:1 ratio. Three DMEM solutions containing different glucose and calcium concentrations (low glucose, high calcium [LG-HC]; high glucose, high calcium [HG-HC]; high glucose, low calcium [HG-LC]) were compared in this study. A media supplementation receipt adapted from Bray et al.^12^ was used and consisted of 10% fetal bovine serum (FBS), 0.1% penicillin/streptomycin solution, 1% non-essential amino acids, 400 mM L-Glutamine, 6.8 mg 3,3,5-triiodo-L-thyronine sodium salt (T3), 1 μg/mL Insulin, 180 μM Adenine, 10 ng/mL epidermal growth factor (EGF), 1 μg/mL isoproterenol, 0.4 μg/mL hydrocortisone, and 5 μg/mL Transferrin. Cells were also grown in the suppliers recommended media, KGM-2 (commercial media). The table given next (Table 1) outlines the different calcium and glucose concentrations for each group.

**Table 1.** Glucose and Calcium Concentrations of the Different Media

Media	LG-HC	HG-HC	HG-LC	KGM-2 media (commercial)
Calcium concentrations (mM)	1.896	1.896	0.096	0.06
Glucose concentrations (mM)	5.5	25	25	6

HG-HC, glucose, high calcium; HG-LC, high glucose, low calcium; KGM-2, keratinocyte growth medium-2; LG-HC, low glucose, high calcium.

### Cell growth curve

After seeding cells in all media formulations, cell growth was measured every day for 7 days in each group. Cells were detached by using trypsin from three separate wells for each group, centrifuged, suspended in media, and counted by using a hemocytometer. Trypan blue exclusion was used to determine the number of cells per milliliter suspension.

### Metabolic activity

A PrestoBlue assay (Thermo Fisher Scientific) was used to assess metabolic activity of cells after 7 days in all groups. This commercially available kit was used following the manufacturer's instructions. Media were aspirated from each well, and a 1:10 mixture of PrestoBlue reagent to media was prepared and added to wells. This was incubated at 37°C for 1 h; the reagent and media were placed in triplicate into a 96-well plate as well as a blank; and absorbance was read at a wavelength of 570 nm.

### RT-PCR

RT-PCR was performed by using a similar protocol to those previously described.^13–15^ RNA was isolated from monolayer cultures by using Trizol (Invitrogen). One milliliter of Trizol was added per well (on a six-well plate) followed by cell scraping. The Trizol-cell solution was collected in RNAse-free Eppendorf tubes, snap frozen in liquid nitrogen, and transferred to −80°C for storage until further use. When ready to be used, the solution was placed on ice to allow it to thaw slowly. Two hundred microliters of chloroform was added to each tube and centrifuged at 12,000 *g* at 4°C. RNA located in the upper phase was transferred to a new RNAse-free tube, isopropanol was added at the same volume as well as 4 μL glycoblue to allow visualization of the RNA in the following steps.

The tubes were stored at −20°C overnight, again placed on ice to allow the solution to thaw, and centrifuged at 12,000 *g* at 4°C for 15 min. A visible blue RNA pellet was formed, the supernatant was discarded, and the tubes were dried. One milliliter of 70% ethanol (in RNAse-free water) was added to wash the pellet. Another centrifugation step was performed at 12,000 *g* at 4°C for 15 min, ethanol was removed, and the pellet was air dried. RNAse-free water (11 μL) was used to dissolve the pellet. A NanoDrop-1000 (Thermo Fisher Scientific) was used to quantify RNA yield and purity. Transcription of mRNA to cDNA was performed by using a high-capacity cDNA reverse transcription kit (Invitrogen).

A mastermix was added to 500 ng of RNA and placed in a thermocycler. The following temperature sequence was applied: 10 min at 25°C, 2 h at 37°C, 5 min at 85°C, and 1 min at 4°C. Quantitative PCR was performed with TaqMan reagents, 4.5 μL cDNA, 5 μL TaqMan universal mastermix II, and 0.5 μL primer. The following primers were used: ERK (Hs00385075_m1), CK3 (Hs00365080_m1), NP63 (custom made primer sequence adapted from Robertson et al.),^16^ and GAPDH (Hs02758991_g1). All samples were run in triplicate with a GAPDH housekeeping gene control. Fold change expression was calculated by using the ΔΔCt method.

### Western blot

Western blot analysis was performed on all groups at day 7. The proteins examined included phosphorylated ERK and total ERK as a loading control. Cell lysates were isolated from monolayer culture by using RIPA Lysis buffer with a phosphatase inhibitor cocktail followed by cell scraping. Samples were centrifuged at 13,000 rpm for 5 min, and the supernatant was used for western blotting. Protein concentration was determined by using Pierce BCA protein assay kit (Thermo Fisher) to ensure equal loading of protein across all conditions, which was 7 μg. The cell lysates were subjected to sodium dodecyl sulfate-polyacrylamide gel electrophoresis (SDS-PAGE) by using a 12% pre-cast polyacrylamide gel at 200 V for 40 min in an electrophoresis rig (Bio-Rad).

A molecular-weight ladder was added to each gel, and proteins were transferred to polyvinylidene difluoride membrane via semi-dry transfer (Thermo Fisher). Membranes were blocked in 3% bovine serum albumin (BSA) in tris-buffered saline (TBS) and 1% Tween 20 (pH 7.6) for 1 h. The membranes were incubated with anti-rabbit phospho-p44/42 mitogen-activated protein kinase (MAPK) (Erk1/2) (Thr202/Tyr204) antibody #9101 and p44/42 MAPK (Erk1/2) antibody #9102 (Cell Signaling) at 1:1000 in 3% BSA in TBS and 1% Tween 20 (pH 7.6) overnight at 4°C with agitation to analyze expression of phosphorylated and total ERK protein, respectively.

The membranes were washed three times for 5 min with TBS and 1% Tween 20 followed by secondary antibody incubation for 1 h. Anti-rabbit immunoglobulin G (IgG) and horseradish peroxidase-linked antibody (Cell Signaling) were prepared at 1:2000 in TBS and 1% Tween 20. Membranes were washed three times for 5 min with TBS and 1% Tween 20, developed by using an immunodetection kit (enhanced chemiluminescence western blotting substrate; Thermo Fisher), and developed by using a GelDoc system (Bio-Rad). Densitometry was performed on all blots by using ImageJ software and graphed by using GraphPad Prism 7.

### Immunocytochemistry

To perform immunocytochemical staining, cells were seeded onto cover-slips, which were activated by using HCl treatment followed by phosphate buffered saline (PBS) washing. After 7 days, the cells were fixed by using 4% paraformaldehyde for 15 min at room temperature (RT) followed by rinsing in PBS three times and stored in PBS until they were ready to stain. The cells were blocked and permeabilized by using 2% FBS and 0.5% Triton-X in PBS (blocking buffer) for 30-min RT, followed by primary antibody incubation at 4°C O/N in 1:10 blocking buffer in PBS (antibody buffer).

Antibodies for mouse anti-vinculin antibody ab18058 (Abcam) and anti-p63 (ΔN), poly6190, rabbit polyclonal 619002 (Biolegend) were used at a 1:500 dilution and CK3 K3/K76 antibody (Sigma-Aldrich) at 1:200 dilution. Cells were washed three times with antibody buffer to remove excess primary antibody and incubated with secondary antibody donkey anti-Mouse IgG H&L (Alexa Fluor^®^ 488) (Abcam) for vinculin and CK3 and goat anti-rabbit IgG (H+L) 647 (Abcam) for NP63 for 2 h at RT at twice the primary antibody dilution. Cells stained with NP63 and CK3 were also stained for cellular actin to visualize the cytoskeleton by using Phalloidin-TRITC (Sigma-Aldrich) at 1:1000 for 2 h at RT with the secondary antibody incubation. All groups were also stained for cellular nuclei by using DAPI (Sigma-Aldrich). Cells were imaged by using a confocal microscope.

### Statistics

All data were analyzed by using GraphPad Prism 6. Data are presented as the mean ± standard deviation, and significance is calculated via one-way ANOVA with Post-Tukey test. All data are presented as *n* = 3.

## Results

### Cell growth curve

**FIG. 1.**
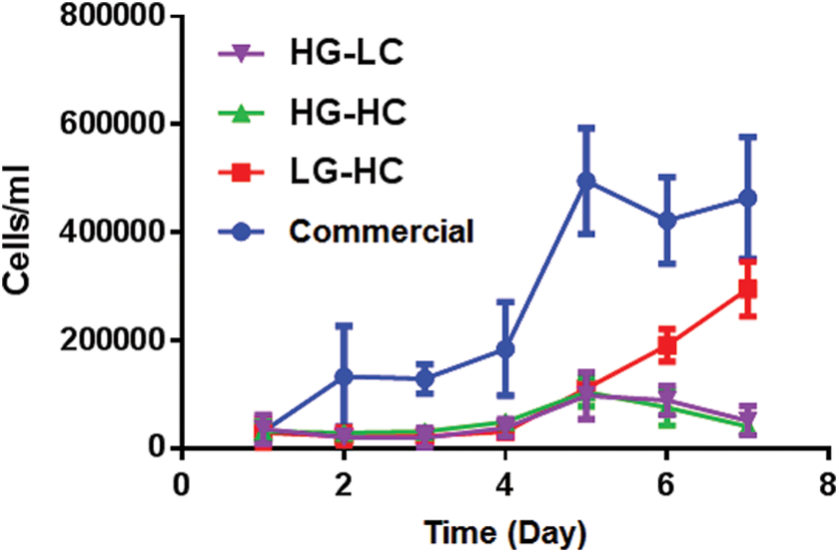
Cell growth curve of hTCEpi cells grown in different media formulations over 7 days. Cells grown in the commercial media displayed a significantly higher cell number over 7 days compared with all other groups. The LG-HC group had a significantly higher rate of growth compared with the HG-HC and HG-LC group. Data are presented as the mean ± SD. HG-HC, high glucose, high calcium; HG-LC, high glucose, low calcium; LG-HC, low glucose, high calcium; SD, standard deviation.

A cell growth curve was prepared over 7 days on all groups, as shown in Figure 1. The commercial group had a significantly higher rate of growth over 7 days compared with all other groups. The cells grown in other media formulations showed a similar rate of cell growth for the first 5 days. The LG-HC group did show an increase in cell growth at days 6 and 7 compared with cells grown in HG-HC and HG-LC. The LG-HC group was found to have a significantly higher rate of growth compared with the HG-HC and HG-LC groups.

### Cell metabolic activity

**FIG. 2.**
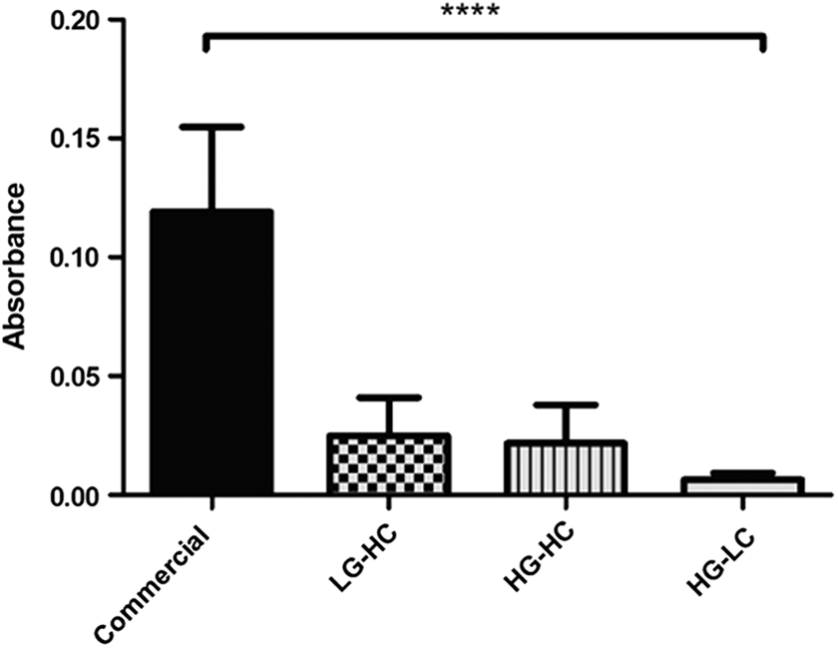
Metabolic activity of hTCEpi cells after 7 days in culture. Cell metabolic activity was significantly decreased in all groups compared with commercial media-cultured cells. Data are presented as the mean ± SD, significance calculated via one-way ANOVA with Post-Tukey test, *n* = 3, ****p ≤ 0.0001. ANOVA, analysis of variance.

A PrestoBlue assay was performed on all groups after 7 days in culture, as shown in Figure 2 to examine the effect that the media formulation had on the cell population's metabolic activity. Cells cultured using the different glucose and calcium concentrations had significantly less metabolic activity compared with the commercial media group. There was no significant difference in metabolic activity between the three test groups. However, the HG-LC group displayed the least metabolic activity of all the groups tested.

### RT-PCR

**FIG. 3.**
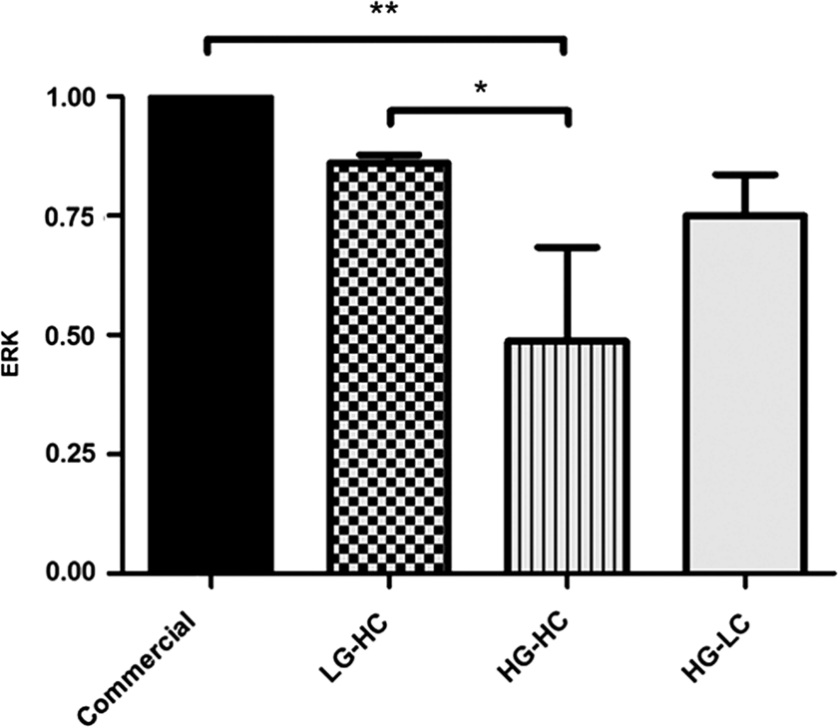
RT-PCR to determine relative expression of ERK after 7 days in culture. HG-HC-fed cells expressed ERK significantly lower than the commercial media-cultured cells and the LG-HC group. Data are presented as the mean ± SD, significance calculated via one-way ANOVA with Post-Tukey test, *n* = 3, *p ≤ 0.005, **p ≤ 0.01. ERK, extracellular-related kinase; PCR, polymerase chain reaction.

Gene expression analysis of the cellular proliferation marker ERK was also performed on all groups after 7 days in culture of the specified culture media (Fig. 3). The HG-HC and LG-HC groups expressed significantly less ERK than the commercial media group. The HG-HC-fed cells expressed ERK that was significantly lower than the LG-HC-fed cells but not the commercial media. The HG-LC group did not show any significant difference in gene expression of ERK when compared with all other groups, including the commercial media.

**FIG. 4.**
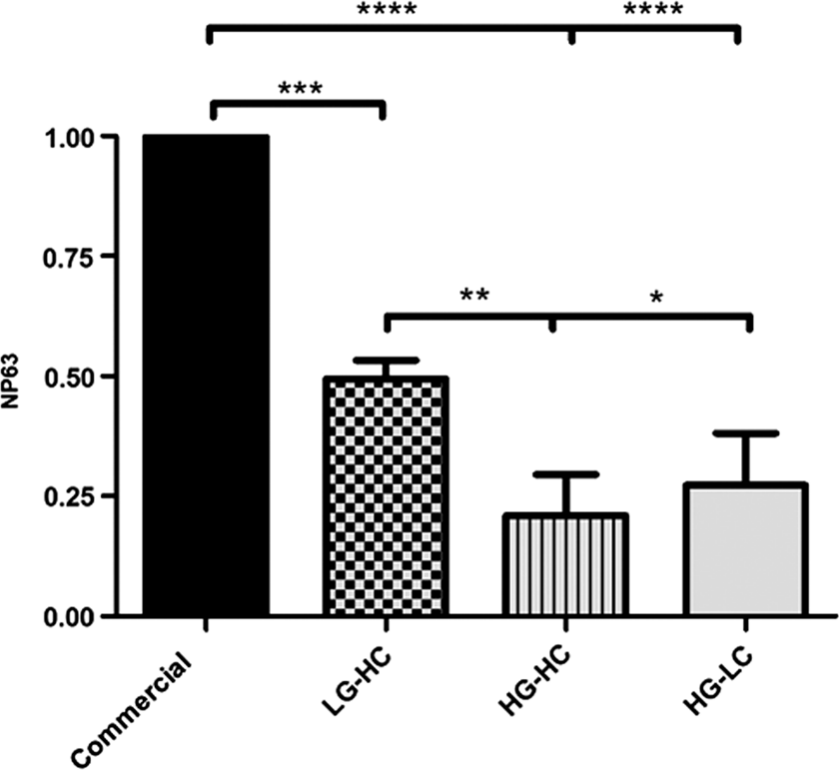
RT-PCR to determine relative expression of NP63 after 7 days in culture. HG-LC and HG-HC groups had the most significant decrease in NP63 expression compared with the commercial media-cultured cells followed by LG-HC. Between groups, the HG-HC group and the HG-LC expressed NP63 significantly lower than the LG-HC group. Data are presented as the mean ± SD, significance calculated via one-way ANOVA with Post-Tukey test, *n* = 3, *p ≤ 0.05, **p ≤ 0.01, ***p ≤ 0.001, ****p ≤ 0.0001.

The corneal epithelial stem cell marker NP63 was analyzed for gene expression after 7 days (Fig. 4). The HG-LC and HG-HC groups showed the most significant decrease in NP63 expression compared with the commercial media group, followed by the LG-HC group. In addition to this, a significant reduction in NP63 expression was observed between groups in the HG-HC group and the HG-LC as well as the LG-HC group.

**FIG. 5.**
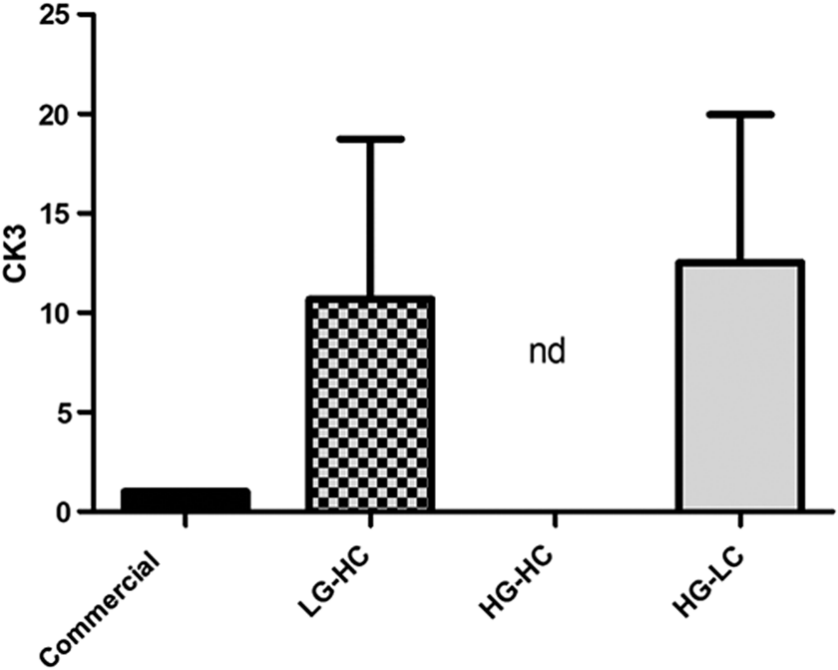
RT-PCR to determine relative expression of CK3 after 7 days in culture. Cells fed in LG-HC and HG-LC media expressed CK3 higher than the commercial media-cultured cells, and CK3 was not detected (nd) in the HG-HC group. No significance was observed between groups or compared with control. Data are presented as the mean ± SD, *n* = 3. CK3, cytokeratin 3.

Gene expression of a mature corneal epithelial marker was also examined (Fig. 5). CK3 was expressed in both the LG-HC and HG-LC groups. However, CK3 was not detected in the HG-HC group. The expression of CK3 was lowest in the commercial media group, but no significant differences were observed either between the groups.

### Western blot

**FIG. 6.**
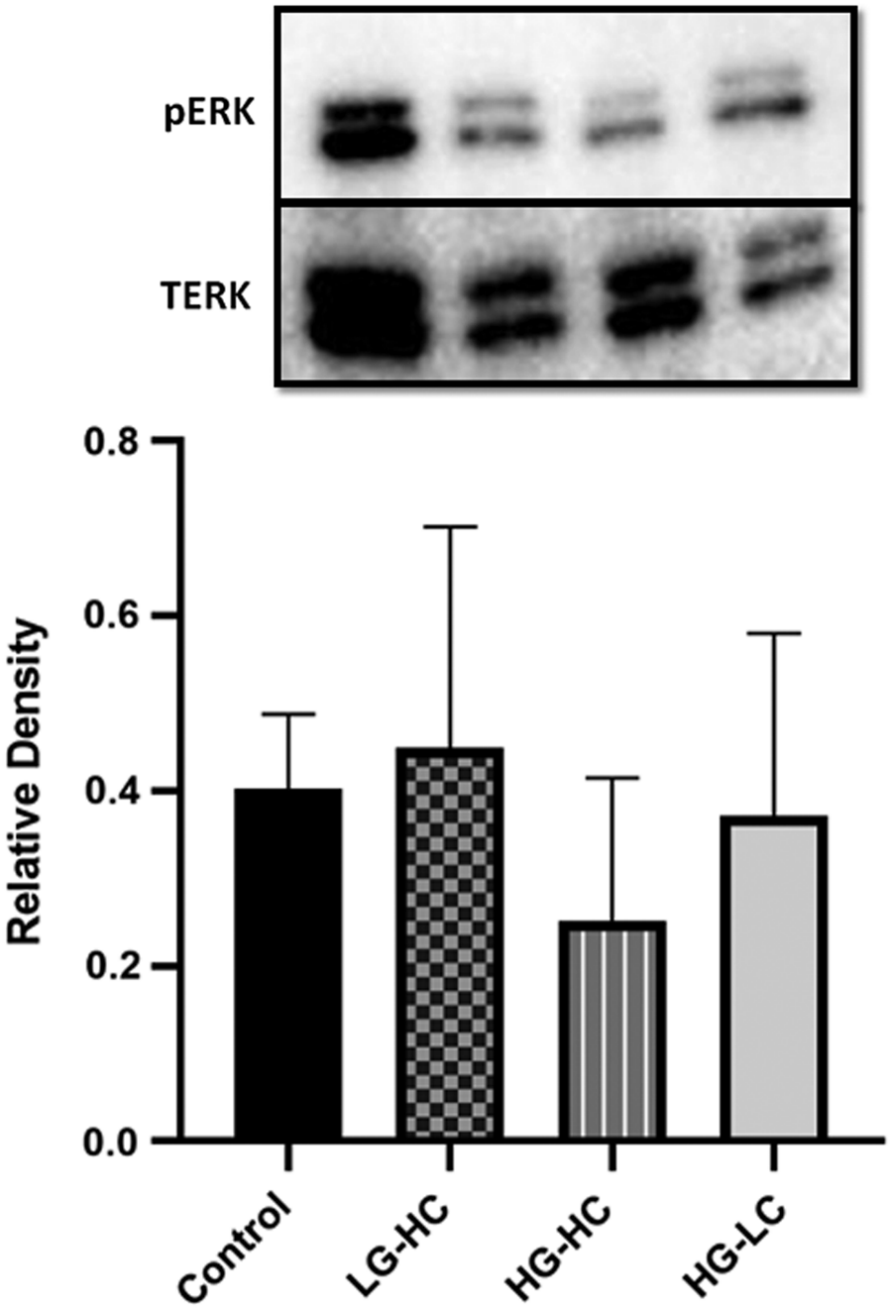
Western blot and densitometry analysis of a corneal epithelial cell line cultured in different media formulations after 7 days. No significant differences between groups were observed of pERK expression. Data are presented as the mean ± SD, *n* = 3.

After 7 days in all media formulations, cells were analyzed for phosphorylated ERK protein expression as a marker for proliferation (Fig. 6). The HG-HC group appeared to express less pERK protein compared with all other groups. The LG-HC and commercial media group produced the highest level of pERK protein, although statistically there was no significant difference in pERK found between any of the groups. When a similar western blot was performed for CK3, there were insufficient proteins detectable to generate a band.

### Immunocytochemistry

**FIG. 7.**
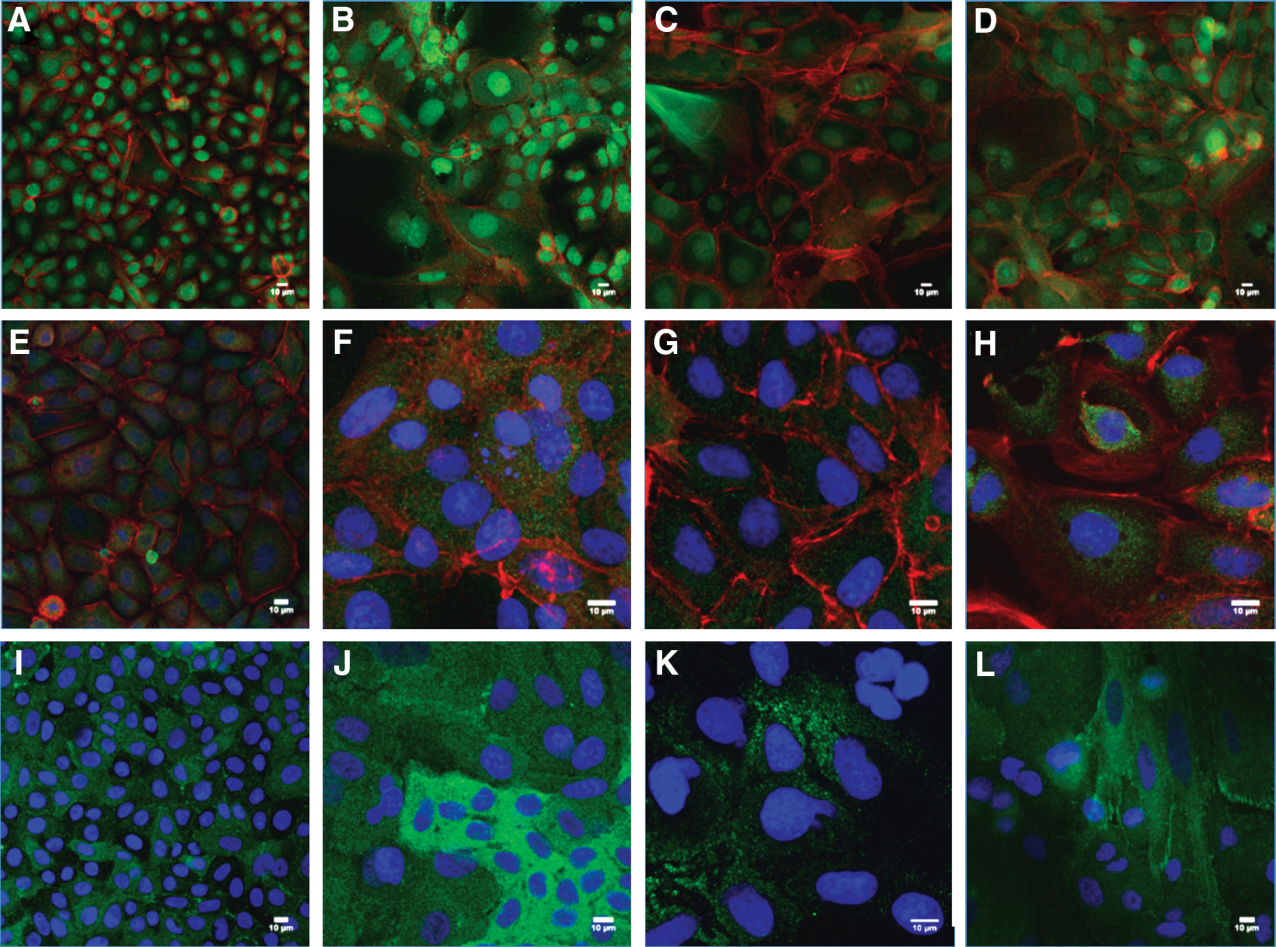
Immunocytochemistry analysis of a corneal epithelial cell line cultured in different media formulations after 7 days. Top row **(A–D)** cells stained in green for NP63 (green); second row **(E–H)** cells stained in green for CK3; and bottom row **(I–L)** cell stained in green for vinculin. All cells were counterstained for f-actin (red) and DAPI (blue). First column **(A, E, I)** shows cells cultured in commercial media; second column **(B, F, J)** shows cells cultured in LG-HC; third column **(C, G, K)** shows cells cultured in HG-HC; and fourth column **(D, H, L)** shows cells cultured in HG-LC (Scale bar = 10 μm). DAPI, 4′,6-diamidino-2-phenylindole.

Cells were analyzed by immunocytochemistry as shown in Figure 7 and imaged by using confocal microscopy at day 7 for stem cell marker NP63 to visualize nuclear localization of the protein as well as mature corneal epithelial marker CK3. In addition to corneal epithelial markers, focal adhesions were examined by using a vinculin stain.

The morphological characteristics of the cells were different between the different groups. In the commercial media group, cells exhibited the typical cobblestone morphology that corneal epithelial cells display with consistent morphology between the cells. Commercial media group cells also were smaller and more densely packed together. In comparison, cells grown using the different test media formulations were more globular and less cobblestone in shape, with some irregular cell shapes present. In particular, LG-HC-fed cells had very irregular cell shapes and adopted a more globular appearance. HG-HC-fed cells were also globular and bigger in size compared with commercial media cultured cells. The HG-LC-fed cells appeared smaller and more regular in cell shape compared with the other test groups.

Nuclear localization of the stem cell marker NP63 was the highest in the commercial media group with little expression in the cytoplasm. Among the test groups, LG-HC-fed cells expressed NP63 the most in the nucleus, with some expression in the cytoplasm. The lowest nuclear expression of NP63 appeared in the HG-HC-fed group. The HG-LC group expressed NP63 in both the nucleus and cytoplasm in similar levels. The mature corneal epithelial cell marker CK3 was least detectable in the commercial media and HG-HC groups. The LG-HC- and HG-LC-fed groups displayed more CK3 with similar expression levels.

Focal adhesions were visualized by using vinculin staining. All groups stained positive for the protein; however, the amount of staining did vary between groups. The commercial media group exhibited an even distribution of staining of the protein over most of the cells, although some cells appeared to show no staining. In the LG-HC group, the intensity of the vinculin stain varied between cells. The HG-LC group also displayed differing amounts of vinculin between different cells but not to the same extent as the LG-HC-fed cells. The HG-HC group had the least vinculin present among all the groups.

## Discussion

Cellular growth was assessed over 7 days in each media formulation by using a trypan blue exclusion assay. It was observed that the commercial media group had a significantly higher level of cell growth compared with all groups. Between groups with varying calcium and glucose concentrations, cellular growth was similar; however, the LG-HC group had a significant increase in cellular growth compared with the other test groups at days 6 and 7, suggesting that calcium increases cellular growth after 5 days.

The metabolic activity of the cells in the different media conditions was examined to provide an indicator of cell proliferation. Although metabolic activity has been previously used to quantify cell proliferation, opinion differs as to how well it truly represents the number of cells present.^17,18^ Although there was no difference between the LG-HC and HG-HC groups, the HG-LC group appeared to demonstrate less metabolic activity, although not significantly different. These results still suggest that calcium concentration plays a role in the proliferation of these cells. Calcium signaling is known to be a key regulator of proliferation for many cell types.^19–21^

A study by Ma and Liu (2011) found that calcium inhibits proliferation and triggers differentiation in mouse corneal epithelial cells,^6^ whereas a study by Kruse and Tseng (1992) showed that the effect of calcium on rabbit corneal epithelial proliferation was dependent on the initial cell seeding density and culture time.^22^ Interestingly, the highest metabolic activity was recorded for the commercial media that had the least calcium. Although this might suggest that low calcium is better for proliferation, it should be noted that other components present in the commercial media differed in concentration or were absent when compared with the test media, and this may also have contributed toward the cells' proliferation.

To further analyze the effect of glucose and calcium on the proliferative capacity of the cells, ERK gene expression was examined. This protein is involved in many cellular processes, including proliferation.^23^ The HG-HC group had a significantly lower expression of ERK compared with LG-HC and commercial media.

In one previous study, high glucose (25 mM) media impaired the EGF receptor (EGFR) signaling pathway in porcine eyes, which is the same pathway that ERK acts on and this delayed epithelial wound healing most likely though ROS.^5^ Interestingly, HG-HC also had a lower expression of ERK compared with HG-LC, which appears to indicate that the combination of HG-HC has a particularly detrimental effect on proliferation. This finding, to our knowledge, has not been previously reported in the literature and should be considered for researchers using this cell line in corneal epithelial research.

Activation of ERK occurs post-transcriptionally via phosphorylation and subsequent translocation to the nucleus, where it activates proliferation. Quantification of western blots found no significant differences between groups; however, HG-HC clearly displayed the least pERK. The LG-HC had a higher expression of pERK compared with the commercial media group. This trend was similar to ERK gene expression and the cell growth curve, showing a significant increase in proliferation after 5 days in the LG-HC group. This may have been due to the observed contact inhibition of the cells grown in the commercial media in which the EGFR pathway has been implicated. Contact inhibition occurs when cells cease proliferation once confluency has been achieved, which is important for tissue homeostasis.^24^ Therefore, it is not surprising that due to the confluency observed in the commercial media-fed cells that pERK is downregulated.

Stem cell marker NP63 was examined by both RT-PCR and immunocytochemistry. This is a well-established marker of a corneal epithelial stem cell phenotype and is present in the limbus of the cornea where stem cells reside.^25^ The highest gene expression was in the commercial media group, indicating that this media retains its stem cell phenotype. This was confirmed by immunocytochemistry analysis where the commercial media group has almost exclusively nuclear localization of the protein. In the test groups, NP63 gene expression was significantly lower than the commercial media group. The most significant decrease in NP63 gene expression and nuclear staining was the HG-HC group. This result shows that HG-HC both inhibits proliferation and causes the cells to lose their stem cell phenotype.

CK3 was used to determine whether the cells were differentiating toward a mature epithelial cell phenotype. It has been shown that CK3 is present only in the suprabasal and superficial cells, indicating that these cells have undergone terminal differentiation.^26^ HG-HC cells did not produce any gene expression for CK3, and it was not visible after staining. This result may indicate that the cells grown in this media formulation begin to differentiate toward an alternative cell phenotype.

Gene expression of CK3 was higher in LG-HC and HG-LC cultured cells compared with commercial media-cultured cells. Immunocytochemistry analysis of these groups showed a similar result, with the commercial media group displaying little staining for CK3 whereas both the LG-HC and HG-LC groups displayed higher levels of the protein. When combined with the data for NP63, these results show that although the commercial media are more beneficial for maintaining a stem cell phenotype, the LG-HC and HG-LC media were better at promoting mature epithelial differentiation.

Vinculin is a focal adhesion protein that is associated with cell signaling, spreading, and migration^27^; hence, it was decided to examine its localization by using immunocytochemical staining. For the corneal epithelium, focal adhesion sites are important to allow the cells to migrate and repopulate regions for both homeostatic and wound-healing responses.^28^ Previous studies have shown that vinculin becomes polarized within corneal epithelial cells to allow migration in a specific direction^29,30^; however, in our study, the staining was more homogeneous within individual cells, although the total amount of vinculin present varies from cell to cell depending on the media used. The confluency of the cells may be a factor in the distribution of vinculin in our study since this would have limited movement of the cells.

The HG-HC group had the least vinculin staining of all the groups. High glucose levels have previously been associated with inhibition of focal adhesions for some cells types,^31^ whereas calcium can also affect vinculin localization and density.^32^

The morphology of the cells was also very different compared with the cells grown in the commercial media. In the control group, cells exhibit typical cobblestone morphology with consistent cell shape and number whereas the test groups had a far more globular appearance with a lower cell number and high nucleus-to-cytoplasm ratio. Cell shape was not consistent and some cells were a lot larger than others, which has been observed in other papers that have investigated calcium concentrations in murine corneal epithelial cells.^6^

## Conclusion

This study showed that the combination of different glucose and calcium concentrations can affect the metabolic activity, proliferative capacity, differentiation, and focal adhesion of a corneal epithelial cell line. The cell line suppliers recommended media appeared to be the best at maintaining stem-like characteristics while also promoting proliferation. Both LG-HC and HG-LC media had reduced expression of NP63 and enhanced expression of CK3, suggesting that these media formulations may be useful for promoting differentiation toward a mature epithelial phenotype. HG-HC media should be avoided when culturing these cells.

## Abbreviations Used

ANOVA: analysis of variance
BSA: bovine serum albumin
CK3: cytokeratin 3
DMEM: Dulbecco's modified Eagle's medium
EGFR: epidermal growth factor receptor
ERK: extracellular-related kinase
FBS: fetal bovine serum
GAPDH: Glyceraldehyde 3-phosphate dehydrogenase
KGM-2: keratinocyte growth medium-2
HG-HC: high glucose, high calcium
HG-LC: high glucose, low calcium
IgG: immunoglobulin G
LG-HC: low glucose, high calcium
MAPK: mitogen-activated protein kinase
PBS: phosphate buffered saline
RT: room temperature
RT-PCR: reverse transcription polymerase chain reaction
SD: standard deviation
SDS-PAGE: sodium dodecyl sulfate-polyacrylamide gel electrophoresis
TBS: tris-buffered saline
